# Diffuse Large B-Cell Lymphoma Masquerading as Recurrent Pleural Effusion and Melena: A Case Report and Literature Review

**DOI:** 10.7759/cureus.111085

**Published:** 2026-06-18

**Authors:** Priyal Mehta, Smitesh Padte, Udaya Kumar Damodaran, Aparna Parvathaneni, Jeffrey Scott, George Abraham

**Affiliations:** 1 Internal Medicine, Saint Vincent Hospital, Worcester, USA; 2 Internal Medicine, Wellspan York Hospital, York, USA; 3 Pulmonary and Critical Care Medicine, Saint Vincent Hospital, Worcester, USA

**Keywords:** acute gi bleed, diffuse large b cell lymphoma (dlbcl), extra nodal non hodgkin lymphoma, rare case report, recurrent pleural effusion

## Abstract

Diffuse large B-cell lymphoma (DLBCL) is the most common subtype of non-Hodgkin lymphoma, accounting for roughly one-third of adult cases worldwide. Typically, patients have a rapidly enlarging mass at presentation, which could be a lymph node or mass lesion anywhere in the body. However, extranodal manifestations and B symptoms can also be observed, and often two-thirds of the patients already present as advanced-stage DLBCL. Early diagnosis of the disease is essential, as early initiation of treatment for advanced stages gives a five-year survival rate of 50-60%. Therefore, it is imperative to recognize unusual presentations of DLBCL and include it in our differential in patients with uncommon extranodal manifestations. We report the case of a 76-year-old man who presented with recurrent pleural effusion and gastrointestinal bleeding and was ultimately diagnosed with multisite extranodal DLBCL involving the stomach, liver, and pleura. The initial presentation raised concerns for primary thoracic or gastrointestinal malignancy, contributing to diagnostic uncertainty. Early recognition of atypical presentations is essential to avoid delays in diagnosis and facilitate timely treatment.

## Introduction

Diffuse large B-cell lymphoma (DLBCL) is the most common histologic type of non-Hodgkin lymphoma, with a male predominance and the median age of presentation being around 64 years [1]. It constitutes a biologically heterogeneous and clinically aggressive malignancy arising from mature B lymphocytes, which, when untreated, progresses rapidly. Although DLBCL classically presents with nodal enlargement, extranodal disease is common and may occur in up to 40% of patients at the time of diagnosis [1]. These extranodal manifestations contribute to the diagnostic complexity of the disease, as multi-organ involvement frequently mimics primary malignancies or inflammatory conditions affecting the same systems.

The gastrointestinal (GI) tract represents the most frequently involved extranodal site, with the stomach accounting for the majority of cases [2]. Endoscopic findings are variable and often nonspecific, ranging from superficial mucosal abnormalities to infiltrative masses or ulcerative lesions. Since these lesions can resemble peptic ulcer disease or primary gastric carcinoma, the diagnosis frequently depends on histopathologic confirmation rather than characteristic endoscopic appearance alone [2]. GI bleeding, obstruction, and perforation are recognized complications, though they remain relatively uncommon initial manifestations [3].

Serous effusions represent another recognized, though diagnostically challenging, manifestation of lymphoma. Pleural, pericardial, and peritoneal effusions may develop through direct tumor infiltration or secondary lymphatic obstruction [4-6]. In patients with non-Hodgkin lymphoma, pleural effusions occur in approximately 6-20% of cases and are frequently lymphocyte-predominant and exudative in nature [7]. Cytologic interpretation of such effusions can be difficult because reactive lymphocytosis may closely resemble malignant lymphomatous involvement, and initial fluid analyses may therefore be nondiagnostic. In these situations, ancillary testing or biopsy of an accessible site, like a pleural biopsy or thoracoscopic sampling, is often required to establish the diagnosis [7].

While extranodal involvement in DLBCL is common, presentations with a constellation of recurrent lymphocyte-rich pleural effusions, GI ulceration, and visceral lesions are distinctly uncommon and may initially suggest metastatic carcinoma, mesothelioma, or infectious etiologies rather than a hematologic malignancy. Awareness of these atypical patterns of disease is important, as delayed recognition may postpone definitive diagnosis and treatment of an otherwise aggressive but potentially treatable lymphoma. Hence, in such patients, an underlying lymphoproliferative disorder should remain an important diagnostic consideration. We report such a case of a patient with disseminated DLBCL whose initial presentation with recurrent lymphocyte-predominant loculated exudative pleural effusions, hepatic nodules, and multifocal gastric and duodenal ulceration complicated by GI bleeding created a challenging diagnostic picture before histopathologic confirmation of lymphoma.

## Case presentation

A 76-year-old male with a history significant for hypertension, hyperlipidemia, hypothyroidism, non-insulin-dependent type 2 diabetes mellitus, gastroesophageal reflux disease (no prior endoscopies available), and being a life-long non-smoker presented to the emergency department (ED) from home after experiencing nausea and lightheadedness. He noted an unintentional 20 lbs weight loss over the prior six months, poor appetite, dyspnea on exertion, and an occasional cough. He denied fever or night sweats, but later, during his hospitalization, he reported experiencing black, tarry stools for over 10 days prior to admission. He had undergone two therapeutic thoracenteses prior to his hospitalization for a large left-sided pleural effusion with removal of 1.8 liters and 1.6 liters 10 days apart. The pleural fluid analysis revealed an exudative and lymphocyte-predominant fluid. Cytology from the initial thoracentesis was negative for malignancy, while flow cytometry revealed polytypic B cells, normal immunophenotypic T cells, absence of immature myelomonocytic cells, and increased natural killer (NK) cells comprising approximately 42% of gated lymphocytes. Pleural fluid analysis from the second thoracentesis revealed a lactate dehydrogenase (LDH) of 288 U/L, protein of 3.4 g/dL, glucose of 119 mg/dL, and no aerobic growth. An outpatient computed tomography (CT) scan of the chest, abdomen, and pelvis was recommended due to recurrent pleural effusions, which demonstrated a large loculated left-sided pleural effusion with associated atelectasis; a left upper lobe nodule measuring up to 14 mm; a peribronchial opacity extending from the left hilum; and multiple hypodense liver lesions described as nonspecific but possibly metastatic. The representative images of the CT scan of the chest, as well as the abdomen and pelvis, are shown in Figure 1.

**Figure 1:**
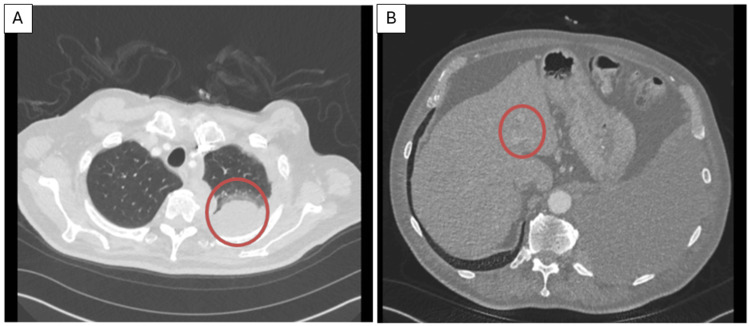
CT chest, abdomen, and pelvis A: CT of the chest showing left-sided loculated pleural effusion with adjacent atelectasis and developing pneumonia. B: CT of the abdomen and pelvis showing multiple hypodense liver lesions. CT: computed tomography.

At admission, he was tachycardic but hemodynamically stable. Labs were notable for mild leukocytosis, normocytic normochromic anemia, blood urea nitrogen of 32 mg/dL, corrected calcium of 11.0 mg/dL, lactate of 1.8 mmol/L, and elevated inflammatory markers (C-reactive protein, erythrocyte sedimentation rate). A chest radiograph revealed a moderate, loculated, left pleural effusion with associated basilar opacities concerning adjacent atelectatic changes. An additional left medial apical mass measuring 4.1 × 2.2 cm with adjacent pleural thickening along the left upper lateral aspect of the lung was noted. A repeat CT angiography was obtained due to an elevated d-dimer and tachycardia, which showed no pulmonary embolism. However, it did show a moderate-to-large loculated left pleural effusion with a 15 mm nodular density in the left upper lobe and a 3 cm perihilar nodular density. It also noted prominent mediastinal lymph nodes, including an 11 mm node in the anteroposterior (AP) window. The patient was initiated on intravenous vancomycin and ceftazidime empirically, for a presumed co-existent bacterial pneumonia. The patient underwent placement of a left-sided PleurX® catheter during the course of his hospitalization due to persistent symptoms and rapid reaccumulation. Post-procedure chest radiograph is shown in Figure 2. Pleural fluid cytology was suspicious for malignancy. Pleural biopsy was considered during hospitalization. However, once endoscopic biopsies and liver biopsy provided diagnostic tissue confirming large B-cell lymphoma, an additional pleural biopsy was no longer required.

**Figure 2:**
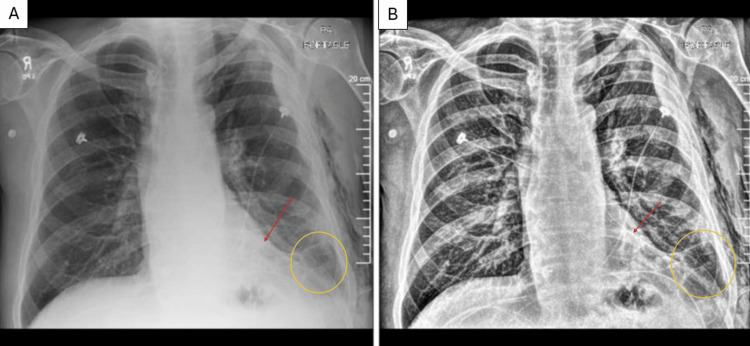
Portable chest radiograph and the corresponding inverted-view image post-procedure (A) Portable anteroposterior chest radiograph showing a moderate left pleural effusion with associated left basilar opacity (yellow circle) and PleurX® catheter (red arrow). (B) Corresponding inverted-view image highlighting the same abnormalities. Yellow circles indicate the left basilar opacity, and red arrows indicate the PleurX® catheter.

The hospital course was notable for melena and acute blood-loss anemia. His hemoglobin trended from 11.4 g/dL on presentation to 8.2 g/dL over the course of three days, with persistent sinus tachycardia and worsening lactate, requiring 3 units of packed red blood cell transfusion over the next 2 days. Esophagogastroduodenoscopy was performed three days after admission, which showed multiple gastric ulcers, including a very large ulcer in the incisura, a moderately large ulcer with smooth margins in the fundus, and multiple smaller ulcers seen in the gastric body. In the duodenum, a larger ulcer was noted. Lesions had raised and smooth borders, but no actively bleeding vessels were noted. Given the multifocal ulcerative lesions, serum gastrin was measured, which was normal. An ultrasound-guided liver biopsy was then performed, as planned tissue sampling was temporarily deferred following the development of acute GI bleeding with symptomatic anemia requiring transfusion. Histopathology and immunochemistry of the pleural fluid cytology, biopsies obtained from the ulcer, and liver core biopsy are shown in Table 1 as well as Figure 3. A summary table for all laboratory values along with reference parameters is available in Table 2.

**Table 1:** Histopathological and immunochemical findings from various biopsy sites Pleural fluid cytology demonstrated rare large atypical lymphocytes with scant CD20-positive large B lymphocytes, numerous CD3-positive T lymphocytes, and scant CD10-positive large B lymphocytes with increased Ki-67 proliferation index. Gastric ulcer margin biopsy revealed infiltration of the gastric mucosa by large, discohesive malignant cells with crush artifact and necrosis. Immunohistochemistry showed positivity for CD20, BCL2, BCL6, and CD10, with a Ki-67 proliferative index greater than 90%, while stains for CAM5.2, CD3, CD5, CD23, cyclin D1, and CD30 were negative. Liver biopsy demonstrated sheets of large, discohesive malignant cells with high nuclear-to-cytoplasmic ratio, brisk mitotic activity, necrosis, and focal crush artifact, consistent with lymphoma. Tumor cells were diffusely positive for CD20 and negative for CD3 and CAM5.2. FISH demonstrated BCL6 rearrangement positivity, while MYC and BCL2 rearrangements were negative. Special stains including *Helicobacter pylori* immunohistochemistry and EBER in situ hybridization were negative. CD20: cluster of differentiation 20; CD3: cluster of differentiation 3; CD10: cluster of differentiation 10; Ki-67: antigen Kiel-67; BCL2: B-cell lymphoma 2; BCL6: B-cell lymphoma 6; CAM5.2: cytokeratin marker; CD5: cluster of differentiation 5; CD23: cluster of differentiation 23; CD30: cluster of differentiation 30; FISH: fluorescence in situ hybridization; MYC: MYC proto-oncogene; EBER: Epstein-Barr virus-encoded RNA.

Biopsy site	Histopathological findings	Immunochemistry	Special stains
Pleural fluid cytology	Rare large, atypical lymphocytes	Scant CD20-positive large B cells, numerous small CD3-positive T lymphocytes, and scant CD10-positive large B lymphocytes, which were positive for Ki-67.	–
Gastric ulcer Margins	Infiltration of the gastric mucosa by large, discohesive malignant cells with crush artifact and necrosis	Positive for CD20, BCL2, BCL6, and CD10. Negative for CAM5.2, CD3, CD5, CD23, cyclin D1, and CD30. Ki-67 was >90%	*Helicobacter pylori* immunohistochemical stain was negative
Liver	Sheets of large, discohesive malignant cells with high nuclear-to-cytoplasmic ratio, brisk mitoses, necrosis, and focal crush artifact, which are consistent with lymphoma	Diffusely positive for CD20. Negative for CD3 and CAM5.2. BCL6 rearrangement positive. MYC and MCL2 rearrangement negative	EBER in situ hybridization stain showed no nucleic acid-staining cells (no EBV infection detected)

**Figure 3:**
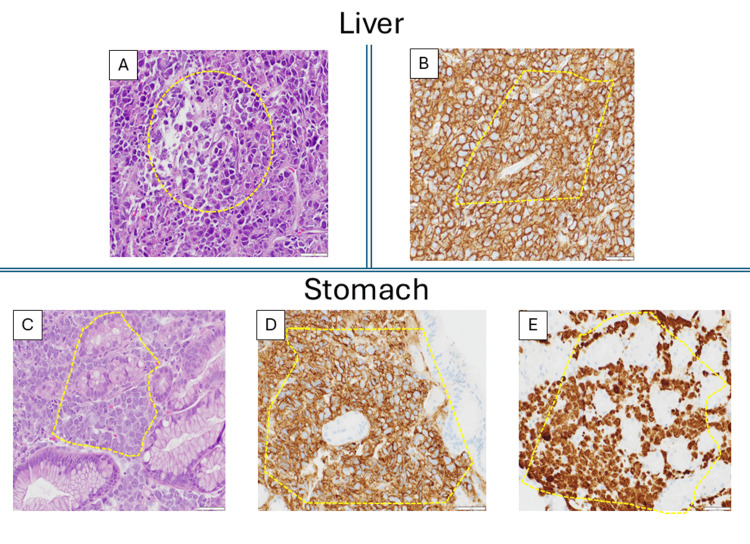
Histopathological and immunohistochemical evaluation of liver and stomach biopsy specimens (A) Liver biopsy, H&E stain at 40× magnification demonstrating diffuse infiltration by large atypical lymphoid cells with marked pleomorphism, irregular nuclei, and high nuclear-to-cytoplasmic ratio. (B) Liver biopsy showing diffuse strong CD20 membranous positivity within the atypical lymphoid infiltrate, consistent with B-cell lineage. (C) Gastric biopsy, H&E stain at 40× magnification demonstrating infiltration of the gastric mucosa by atypical lymphoid cells involving the glandular epithelium. (D) Gastric biopsy with diffuse CD20 positivity in infiltrating tumor cells. (E) Gastric biopsy demonstrating Ki-67 proliferative index greater than 90% within tumor cells, indicating a highly proliferative neoplasm. Yellow dashed outlines highlight representative areas of abnormality. H&E: hematoxylin and eosin; CD20: cluster of differentiation 20; Ki-67: antigen Kiel-67. Image credits: Authors compiled using Microsoft PowerPoint® (Microsoft, Redmond, WA, USA).

**Table 2:** Laboratory and pleural fluid analysis findings Reported serum, cerebrospinal fluid, and pleural fluid laboratory values during the diagnostic evaluation, including reference ranges and interpretation where applicable. Reference ranges are provided as standard adult ranges and may vary by institutional laboratory. LDH: lactate dehydrogenase; BUN: blood urea nitrogen.

Parameter	Reported value	Reference range/Normal value	Interpretation
Pleural fluid LDH	288 U/L	Variable; interpreted relative to serum LDH (Light's criteria)	Consistent with exudative effusion in clinical context
Pleural fluid protein	3.4 g/dL	Variable; interpreted relative to serum protein (Light's criteria)	Supports exudative effusion
Pleural fluid glucose	119 mg/dL	70-140 mg/dL	Within expected range
Pleural fluid culture	No growth	No growth	Negative culture
Natural killer cells in pleural fluid	~42%	Typically 5-20% of circulating lymphocytes	Increased
BUN	32 mg/dL	7-20 mg/dL	Elevated
Corrected calcium	11.0 mg/dL	8.5-10.5 mg/dL	Mild hypercalcemia
Lactate (Admission)	1.8 mmol/L	0.5-2.0 mmol/L	Normal
Hemoglobin (Presentation)	11.4 g/dL	Male: 13.5-17.5 g/dL	Anemia
Hemoglobin (Nadir)	8.2 g/dL	Male: 13.5-17.5 g/dL	Severe anemia
Serum LDH	602 U/L	140-280 U/L (laboratory dependent)	Elevated
Cerebrospinal fluid flow cytometry	Negative for malignant cells	Negative	Normal finding
Serum gastrin	79 pg/mL	Typically <100 pg/mL	Not suggestive of Zollinger-Ellison syndrome

A diagnosis of diffuse large B-cell lymphoma of the germinal center B-cell phenotype was made with involvement of the stomach, liver, pleura, and possibly the lungs. The patient was staged as Ann Arbor stage IV. LDH was 602 U/L. Eastern Cooperative Oncology Group (ECOG) performance status was 2, National Comprehensive Cancer Network-International Prognostic Index (NCCN-IPI) score was 7, and Central Nervous System-International Prognostic Index (CNS-IPI) score was 5. CSF flow cytometry was negative for malignant cells. The patient was initiated on rituximab, cyclophosphamide, doxorubicin, vincristine/oncovin, and prednisone (R-CHOP) chemotherapy within 10 days of initial presentation. The first cycle was well tolerated aside from mild laboratory tumor lysis syndrome. Ongoing management plans include intermittent catheter drainage three times a week, proton pump inhibitors and sucralfate for the gastroduodenal ulcers, and interval PET/CT following three cycles of chemotherapy. The timeline of management is demonstrated in Figure 4.

**Figure 4:**
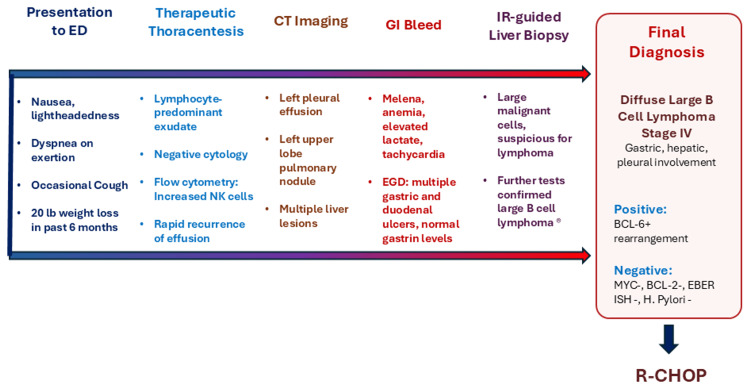
Diagnostic timeline illustrating the clinical course and stepwise evaluation leading to the diagnosis of advanced DLBCL DLBCL: diffuse large B-cell lymphoma; ED: emergency department; CT: computed tomography; GI: gastrointestinal; NK cells: natural killer cells; EGD: esophagogastroduodenoscopy; BCL-6: B-cell lymphoma 6; MYC: MYC proto-oncogene; BCL-2: B-cell lymphoma 2; EBER ISH: Epstein-Barr virus-encoded RNA in situ hybridization; *H. pylori*: *Helicobacter pylori*; R-CHOP: Rituximab, Cyclophosphamide, Doxorubicin, Vincristine, and Prednisone. Image credits: Authors using Microsoft PowerPoint® (Microsoft, Redmond, WA, USA).

## Discussion

This case illustrates a diagnostic challenge posed by DLBCL, in which the initial presentation was recurrent unilateral lymphocyte-predominant pleural effusion, with only extrathoracic tissue from malignant- appearing gastroduodenal ulcers and hepatic lesions yielding a conclusive diagnosis. This sequence is clinically relevant because DLBCL represents the most common subtype of NHL seen in adults; however, it has diverse presentations in terms of anatomic distribution and histopathologic characteristics. Moreover, extranodal involvement can challenge clinicians when it masquerades as a primary thoracic or GI disorder. Recent literature reviews suggest that DLBCL should not be considered a single clinicopathologic entity but rather a spectrum of aggressive large B-cell neoplasms originating from either nodal or extranodal tissues, requiring correlation of clinical, morphologic, immunophenotypic, and molecular data for proper risk assessment [8-10].

The pleural component of our case was especially instructive. Although lymphomatous effusions are not uncommon, lymphocyte predominance poses a cytopathologic dilemma, given a significant overlap between reactive and neoplastic processes. In fact, Koh J et al. highlight that pleural effusions are seen in 6-20% of patients with NHL [7]. Diffuse large B-cell lymphoma is one of the most frequent causes of large-cell lymphoid effusions, and morphologic assessment should be used in conjunction with ancillary studies, including immunocytochemistry, in situ hybridization, clonality studies, and, when appropriate, next-generation sequencing [11]. High cellularity, monomorphism, atypia, mitotic rate, apoptosis, and necrosis are some of the cytomorphologic features that suggest neoplasia vs. a reactive process, with the latter features more commonly seen in polymorphous, small-cell-predominant fluids. Regarding our patient’s course, initial pleural fluid evaluation showed lymphocyte-predominant exudate without definite evidence of malignant cells, and subsequent evaluation of cell block immunostains showed atypical large B cells (positive for CD20 and CD10). This was similar to the diagnostic challenges reported by Chen et al., emphasizing that DLBCL, although rare, should be considered in unresolved isolated lymphocytic pleural effusions [12]. Additionally, like our case, Sivakolundu et al. reported recurrent lymphocyte-rich pleural effusion with a cavitary lung lesion and a heterogeneous liver lesion that was initially treated as an infection, but further testing suggested a primary pulmonary lymphoma [4]. It is thus important to note that thoracic DLBCL can masquerade as pneumonia, organizing infection, or other pleural diseases, posing a diagnostic challenge. On the other hand, Itmam et al. reported that recurrent pleural effusions may appear as a primary thoracic disease, but one should not exclude a second hematologic malignancy, particularly when cytology discloses atypical lymphoid cells [11].

The prognostic significance of effusions in DLBCL also strengthens the importance of this presentation. In the study by Chen et al., 18.4% of patients with DLBCL had effusions, and malignant effusions correlated with higher stage, higher IPI, and worse outcomes [13]. Notably, malignant effusion was described as a poor prognosticator and, in their analysis, even worse than stage IV disease alone [14]. Duminuco and her team concluded that patients with lymphomatous effusions should be treated as having stage IV disease and that systematic analysis of effusions for tumor cells should be part of the routine workup. In our patient, later pleural cytology was suspicious for large B-cell lymphoma, which aligned with the advanced extranodal distribution already established by gastric and liver biopsies.

A multicentric 2023 GI lymphoma study showed that the stomach is the most common site of GI lymphoma [2]. Aggressive lymphomas more commonly have fungating or protruding ulcerative lesions, but the endoscopic appearance is often nonspecific and, thus, histopathology remains indispensable for diagnosis. Another study by Kyaw et al. demonstrated that GI bleeding is a presenting symptom in 20-30% of gastric DLBCL [3]. It also highlights that distinguishing poorly differentiated gastric carcinoma from gastric lymphoma can be difficult on endoscopic biopsies and may require expanded immunohistochemistry. Similarly, a case report by Andrews et al. describes high-grade B-cell lymphoma initially resembling ovarian malignancy with bilateral ovarian masses, omental disease, ascites, and markedly elevated CA-125 levels, with flow cytometry and FISH clarifying the diagnosis [6]. Hence, our patient’s EGD is of particular significance for understanding the complexity of DLBCL. It demonstrated multiple gastric ulcers and duodenal ulcers with raised margins, which were suspicious of malignancy. However, the diagnosis depended on a biopsy showing large B-cell lymphoma with advanced immunohistochemistry with CD20, CD10, BCL6, and BCL2 expression and a Ki-67 >90%.

A 2025 review indicates that workup for the disease should include tissue acquisition for histopathology and immunophenotyping, FISH testing for MYC (with additional BCL2/BCL6 testing when clinically indicated), PET/CT scanning for staging, and thorough bloodwork with viral serologies [14]. Furthermore, the review emphasizes that a core-needle biopsy, performed with image guidance, is a safe alternative capable of providing adequate tissue for comprehensive histologic, immunophenotypic, and molecular evaluations, especially when an excisional biopsy is not an option. In our case, liver biopsy proved to be a key to diagnosis, followed by FISH analysis showing a BCL6 rearrangement but no MYC or BCL2 involvement and a negative EBER ISH. As stated by Duminuco and team, immunohistochemical algorithms like Hans can still be used as a surrogate marker for cell of origin, which, in our case, was consistent with the germinal center B-cell phenotype. Hence, the diagnostic and staging approach in this instance adhered to current best practice guidelines, which are especially important for prognosis. In that regard, the NCCN-IPI retains its utility, as it considers age, LDH, performance status, stage, and number of extranodal regions involved to better identify high-risk patients than the original IPI [1]. Contemporarily, the risk stratification trends are evolving to account for multiple clinical factors alongside molecular subclassification (i.e., cell-of-origin and MYC/BCL2/BCL6 alterations) and emerging biomarkers (i.e., circulating tumor DNA). In terms of imaging, metabolic tumor burden measured by PET-CT is proving to be a significant factor in predicting patient outcomes [14]. According to current protocols, while interim PET scans do retain a role in predicting outcomes, their use is best reserved for risk stratification and not for modifying treatment regimens. Our case featured Ann Arbor stage IV disease with elevated LDH, four risk-enhancing extranodal sites (stomach, liver, pleura, and likely lung), an ECOG performance status of 2, and elevated NCCN-IPI and CNS-IPI scores. This constellation of characteristics is consistent with biologically aggressive and clinically high-risk DLBCL.

With respect to treatment, management was guided by the current standard therapeutic approaches. As outlined by Sehn et al., anthracycline-based chemoimmunotherapy with R-CHOP remains the first-line treatment for most patients with advanced-stage disease, resulting in durable remission in about 60% of patients [1]. Ansell and Nowakowski underlined the latest therapeutic approaches available in 2026 for DLBCL [15]. Strategies to intensify treatment have included dose-dense approaches as well as DA-EPOCH-R, but neither has demonstrated superiority when compared with R-CHOP in an unselected patient population while being associated with higher rates of toxicity [16]. However, there are now risk-adaptive approaches that allow for the intensification of neoadjuvant treatment. Polatuzumab vedotin (Pola) combined with R-CHP (Pola-R- CHP) was recently shown to improve progression-free survival (PFS) without a significant overall survival (OS) benefit [17]. In patients harboring high-risk molecular features such as MYC and BCL2 rearrangements, intensified regimens like lenalidomide plus DA-R-EPOCH may be considered [18]. For relapsed or refractory disease, treatment has dramatically changed over the last few years. CD19-directed CAR T-cell therapy now supersedes salvage chemoimmunotherapy plus transplant in certain early-relapse or primary refractory cases [19]. Bispecific antibodies and antibody-drug conjugates, on the other hand, provide depth of coverage for later lines of therapy or for patients who are not suitable candidates for cellular therapies [20].

## Conclusions

Our case demonstrates an uncommon, aggressive, and diagnostically challenging diffuse large B-cell lymphoma with concurrent GI, hepatic, and pleural involvement, wherein an initial high index of suspicion led to successful early diagnosis and treatment. A major strength lies in multimodal diagnostic confirmation with tissue histopathology and pleural fluid cytology with immunophenotyping. Limitations are inherent to the constraints of a single case and, thus, response assessment and long-term outcome data remain limited. This case underscores the need to consider lymphoma in the differential diagnosis of persistent lymphocyte-predominant pleural effusions when accompanied by unexplained GI bleeding or visceral lesions. Recognition and diagnosis of this disease should occur expediently with tissue diagnosis, followed by staging studies and institution of systemic therapy. While there are newer agents and approaches being tested and validated, treatment with R-CHOP is still the standard of care for initial therapy. Disease biology and risk stratification continue to play larger roles in prognosis.
